# Clinico-epidemiological characteristics of cerebral venous sinus thrombosis in Kenya: a retrospective case series

**DOI:** 10.3389/fstro.2025.1599755

**Published:** 2025-07-30

**Authors:** Taby Siika, Jaskirat Sokhi, Juzar Hooker, Sheila Waa, Anne Mwirigi, Jasmit Shah, Dilraj Singh Sokhi

**Affiliations:** ^1^Department of Internal Medicine, Aga Khan University, Nairobi, Kenya; ^2^Department of Radiology, Aga Khan University, Nairobi, Kenya; ^3^Department of Oncology/Haematology, Aga Khan University, Nairobi, Kenya; ^4^Brain and Mind Institute, Aga Khan University, Nairobi, Kenya

**Keywords:** Sub-Sahara Africa (SSA), cerebral venous sinus thrombosis, thrombophilia, neuro-imaging, retrospective study

## Abstract

**Background:**

Cerebral venous sinus thrombosis (CVST) is a rare cause of stroke that is more common in young, especially female, adults and can be challenging to diagnose due to its frequently non-specific presentation and diverse risk factors. Most cases are idiopathic, and international guidelines do not recommend routine investigations for underlying thrombophilia. Timely diagnosis, with prompt neuroimaging and guideline-based treatment, leads to good outcomes. However, in the literature on CVST from sub-Saharan Africa, the gap is substantial, with the few cases described as being related to systemic and/or brain infections. We describe here the largest cohort of CVST from the region with novel findings that may be relevant to everyday clinical practice.

**Methods:**

We conducted a retrospective cross-sectional study of patients diagnosed with CVST from 2010 to 2022 at our tertiary regional neurology referral center in Nairobi, Kenya.

**Results:**

We identified 122 cases: 67.2% (82/122) were female, 80.3% (98/122) were Black African, and the median (interquartile range) age was 36.8 (31.5–45.7) years. Apart from headaches (86.9%, 106/122), the most common presenting symptoms were visual disturbance (26.2%, 32/122) and seizures (23.8%, 29/122); 11 patients developed seizures later. Intracranial hemorrhage with and without venous infarction occurred in 27.9% (34/122) of patients. New diagnoses of thrombophilia were made in 30.3% (37/122). Other causes were HIV, hepatitis B/C, or other brain infections (18.0%, 22/118); pregnancy, including postpartum (14.6%, 12/82); contraceptive use (8.5%, 7/82); and malignancy (8.1%, 10/122). The most common treatment was with warfarin in 50% (61/122), followed by rivaroxaban (29.5%, 36/122) and dabigatran [14.8% (18/122)]. Complete thrombus resolution occurred in only 53.9% (55/102) at follow-up scanning (at a median of 178 days). In terms of outcomes (modified Rankin Score [mRS]), 32.8% (40/122) had an mRS score = 0, 59.9% (73/122) had an mRS score = 1–2, and there was one fatality who also had concurrent systemic malignancy.

**Conclusion:**

Thrombophilia was more prevalent in our cohort of CVST than infections, which is a novel finding compared to what has been published about CVST from sub-Saharan Africa. Most patients were managed with appropriate anticoagulants, but only about half the patients had complete resolution of the CVST at last follow-up. We therefore recommend that thrombophilia should be routinely investigated in all patients with CVST in our setting.

## Introduction

Cerebral venous sinus thrombosis (CVST) is a rare cerebrovascular disease caused by partial or complete occlusion of the major intracerebral venous sinuses and/or the smaller feeding cortical veins. Patients usually present with headaches, focal neurological deficits, and seizures, and it is more commonly described in females (Ulivi et al., 2020). Standard diagnostic and management criteria exist (Ferro et al., 2017), with recent updates on the efficacy of novel anticoagulants in managing the condition.

There is a significant paucity of data from sub-Saharan Africa (SSA) and on CVST (Baduro and Ferro, 2021), with cohorts mainly published from the West (Gaye et al., 2021; Napon et al., 2010) and North (Sidhom et al., 2014; Sassi et al., 2017; Lounici et al., 2020) regions. A recent systematic review of data published from SSA concluded that most patients were female, the most common etiology was infections, and prothrombotic conditions were rare (Baduro and Ferro, 2021). Furthermore, non-dedicated computed tomography (CT) scans were the most common modality used, and death rates were higher than in high-income countries. The data in this systematic review were notably sparse from East Africa: case reports from Uganda (Arbela, 1964) and a retrospective review of 51 cases from Kenya (Onyambu et al., 2013), which supported infections, including HIV, being the main risk factor. See Table 1 for a summary of the studies. Few data exist from the region on CVST management and patient outcomes.

**Table 1:** Summary of CVST cases from sub-Saharan Africa.

**References**	**Country**	**Patients**
Mwita et al. (2013); Mokgacha et al. (2017)	Botswana	3
Napon et al. (2010)	Burkina Faso	18
Konin et al. (2008)	Cote d'Ivoire	1
Onyambu et al. (2010, 2013)	Kenya	53
Hunald et al. (2011)	Madagascar	1
Okunola et al. (2012); Makanjuola et al. (2015)	Nigeria	2
Gaye et al. (2021); Dadah et al. (2015)	Senegal	71
Seid and Sellars (1973); Samuel and Fernandes (1987); Urquhart et al. (1989); Singh (1993); Duncan and Fourie (2005)	South Africa	96
Idris et al. (2008)	Sudan	15
Scrimgeour et al. (1991); Kalangu (1995)	Zimbabwe	27
**Total**		**287**

Our tertiary neurology referral center in Nairobi, Kenya, has advanced diagnostic availabilities (magnetic resonance imaging [MRI] with dedicated vascular imaging) and a greater availability of neurologists (6 of the 18 neurologists in Kenya are based at our center) and, therefore, has become a hub for regional referrals of CVST. We describe these cases but further expand on management and outcomes that have, so far, not been described in SSA.

## Materials and methods

### Study design

We conducted a retrospective cross-sectional study of adult (age >18 years) patients admitted to our hospital with CVST from 2010 to 2022 inclusively on patients with CVST who were managed in our hospital. Deidentified data on the patient's clinical presentation, imaging findings, treatment given, and clinical outcomes were directly entered into REDCap^®^.

### Ethical considerations

The Aga Khan University Institutional Scientific and Ethics Review Committee (Ref: 2022-ISERC-126), and the National Committee for Science, Technology and Innovation (NACOSTI, Ref No: 238026) granted ethical approval before the study commenced.

## Results

The results are summarized in Tables 2, 3.

**Table 2:** Sociodemographic, clinical, and investigation characteristics of the study population.

**Variable**	**Sub-variable**	***n* = 122 (%)**
Age (years)	*median (IQR)*	36.8 (31.5, 45.7)
Gender	Female	82 (67.2%)
	Male	40 (32.8%)
Ethnicity	Black African	98 (80.3%)
	South Asian	9 (7.4%)
	Caucasian	4 (3.3%)
Clinical presentation	Headache/Neck ache	117 (95.9%)
	Seizure	32 (28.3%)
	Visual problems	32 (26.2%)
	Vomiting/dizziness	27 (22.1%)
	Focal neurologic deficit	21 (17.2%)
	Altered consciousness	11 (9.0%)
Duration (days)	*median (IQR)*	6.0 (3.0, 17.0)
Comorbidities	Unrelated non-communicable diseases	56 (45.9%)
	Infections	22 (18%)
	Pregnancy (*N* = 82)	12 (14.6%)
	Malignancies	10 (8.2%)
	Oral contraceptive pills (*N* = 82)	7 (8.5%)
	Autoimmune diseases	6 (4.9%)
	Inflammation	5 (4.1%)
	Lumbar puncture	3 (2.4%)
	Surgical procedures	3 (2.4%)
	Head injury	1 (0.8%)
Laboratory investigations (*n* = 106)	Autoimmune panel	87 (82.1%)
	Thrombophilia screen positive	37 (30.3%)
Neuroimaging (MRI +/– CT)	>1 sinus involved	94 (77%)
	Brain oedema	15 (26.8%)
	Venous infarction	29 (51.8%)
	Hemorrhage	34 (60.7%)
	Herniation	4 (7.1%)

MRI, magnetic resonance imaging; CT, computed tomography.

**Table 3:** Treatments and outcome of cerebral venous sinus thrombosis cohort.

**Acute treatment**	**Subcutaneous LMWH**	**121 (99.2%)**
	Fondaparinux	1 (0.8%)
	Decompressive hemicraniectomy	2 (1.6%)
	ASM	32 (28.3%)
Maintenance treatment	ASM duration, months (median [IQR])	16.0 (10.0, 24.0)
	*Warfarin 61 (50.8%)*	*DOAC 60 (49.2%)*
Follow-up imaging^*^	(*n* = 51)	(*n* = 51)
No resolution of clot(s)	4 (7.8%)	10 (19.6%)
Partial resolution	16 (31.4%)	17 (33.3%)
Complete resolution	31 (60.8%)	24 (47.1%)
Not done	20 (16.4%)	
Persistent symptoms at follow-up (*n* = 83)	Headache	64 (77.1%)
	Weakness	13 (15.7%)
	Numbness	2 (2.4%)
	None	39 (32%)
mRS score up to 3 months	No disability (mRS score: 0–1)	89 (81.2%)
	Mild/moderate disability (mRS score: 2–3)	17 (14.0%)
	Moderate/severe disability (mRS score: 4–5)	5 (4.1%)
	Death (mRS 6)	1 (0.8%)

LMWH, low-molecular-weight heparin; ASM, anti-seizure medications; DOAC, direct oral anticoagulants; mRS, modified Rankin Scale. ^*^p= 0.175.

## Discussion

### Demographics and presentation

The majority of our CVST cohort was female and relatively young, comparable to other studies from within and outside SSA (Baduro and Ferro, 2021). More than 80% were of Black African origin, providing adequate representation of the local population.

The top three symptoms at presentation were headache, focal neurological deficits, and seizures, which are, again, comparable to case series from SSA (Baduro and Ferro, 2021). However, we did not find any cases of cavernous sinus syndrome (CSS), which has been more often described, in up to 13.8% of cases, in other SSA cohorts. This could be because CSS is usually infective in nature, and infections were less implicated in our cohort (18% vs. 63.1% for other case series from SSA).

### Risk factors for CVST

When comparing our cohort to other SSA cohorts, frequencies of contraceptive use (8.5% vs. 7.3%), pregnancy- and puerperium-related complications (9.8% vs. 6.2%), and traumatic and iatrogenic (5.6% vs. 0.8%) were not significantly different (Baduro and Ferro, 2021). However, we had more cases attributed to underlying systemic inflammatory/connective tissue diseases (9.0% vs. 3.7%) and malignancies (8.2% vs. 1.5%). Our center is a regional referral hospital for rheumatological and oncological conditions and has international-standard diagnostic services, which may explain this higher level of diagnosis (Pathology Laboratory Medicine, 2015). As has been described (Zhang et al., 2023), CVST with underlying malignancy, especially hematological, was associated with a poorer outcome in our cohort.

We found blood-borne viral infections, including HIV and hepatitis, to be the main infective risk factor for CVST in our cohort, similar to what has been found in other parts of the continent (Baduro and Ferro, 2021; Gaye et al., 2021; Napon et al., 2010) but different from the findings of the other Kenyan study (Onyambu et al., 2013), which was CVST being more common with direct brain infections such as tuberculosis. This within-country difference in the type of infection associated with CVST is likely due to the other Kenyan cohort being from a large public hospital where tuberculosis and other HIV-related opportunistic infections are more common (Chepkondol et al., 2020).

Of considerable significance was the number of patients newly diagnosed with thrombophilia in our cohort (30.3%), with protein S deficiency being the most common diagnosis (16.4%). This is an important finding brought about by routine thrombophilia testing in all CVST patients, contrary to what has been suggested by the latest European guidelines (Ferro et al., 2017). In comparison, the SSA cohorts had thrombophilia detected in 2.2%, with protein S deficiency being slightly less common than anti-phospholipid syndrome (Baduro and Ferro, 2021). The larger North African and Mexican cohorts had similar low findings of thrombophilia (Sassi et al., 2017; Ruiz-Sandoval et al., 2012); our cohort is similar to what has been found in, for example, European studies (Ferro et al., 2004).

### Neuroimaging

A large proportion of our patients (83.6%) had contrast-enhanced MRI brain scan with venography and angiography, compared to 20.5% having contrast-enhanced CT head scans with or without venography. This contrasts significantly with other local studies, where the most common modality was a head CT scan with contrast, rarely with venography, and no MRI scans (Onyambu et al., 2013). This reflects the variability of MR diagnostic services available in private hospitals vs. public hospitals in SSA, including in Kenya (Hasford et al., 2022). The superior sagittal sinus was the dominant sinus involved (51.6%), as is the case with other cohorts (Baduro and Ferro, 2021; Ruiz-Sandoval et al., 2012; Ferro et al., 2004; Duman et al., 2017; Khealani et al., 2008). The most common secondary parenchymal brain lesions were hemorrhage (60.7%) followed by what has usually been reported in other studies: venous infarction (51.8%), brain oedema (26.8%), and herniation (7.1%) (Gaye et al., 2021; Wasay et al., 2019). The higher rates of hemorrhage are probably due to the main non-communicable disease comorbidity being hypertension in our patients (16.4%). Interestingly, patients with hypertension were significantly less likely to have complete resolution of CVST at follow-up imaging (*p* = 0.003), a finding worth exploring in larger future studies to identify the nature behind this relationship.

### Treatment and outcomes

In the acute phase, all our patients were started on low-molecular-weight heparin (LMWH); one was treated with fondaparinux due to associated comorbidities. Slightly more than half transitioned to warfarin, and the remainder were treated with direct oral anticoagulants (DOACs), even though the evidence for DOAC use in CVST has only been published in recent years (Ferro et al., 2019; Field et al., 2023). DOAC preference was probably because the infrastructure for monitoring warfarin treatment in our setting is poor, a systemic problem across SSA (Mouton et al., 2021; Tadesse et al., 2022). Patients from our hospital are more likely to be prescribed DOACs due to a number of factors, including the ability to afford them compared to other hospitals (Mucyo et al., 2022). It is noteworthy that the patients in our study who were managed with warfarin had slightly better clot resolution rates, but this was not statistically significant (*p* = 0.175).

The majority (82.5%, 33/40) of patients who experienced seizures were treated with levetiracetam for a median duration of 16.0 (10.0, 24.0) months, in keeping with international guidelines (Ferro et al., 2017), chiefly to avoid interactions with anticoagulation using other anti-seizure medications. Only 20% of these patients had seizures beyond the first 3 months, as is expected in acute symptomatic seizures. However, headaches remained a significant problem for most patients throughout their treatment (77.1%), even though only a small majority had premorbid primary headache conditions. This is an increasingly recognized phenomenon globally (Dias et al., 2024).

We had a higher proportion of patients having good outcomes compared to other studies from the continent (Baduro and Ferro, 2021; Gaye et al., 2021; Sassi et al., 2017): 92.7% had a modified Rankin Scale (mRS) score of 0–2, of which 81.2% had no disability (mRS score: 0–1) at last follow-up. We suspect this could be because of the ability of our center to reach an early diagnosis compared to other SSA cohorts:

- Our hospital has readily available state-of-the-art diagnostics: In additional to being one of only five sites in the country that have 3T MRI scanners, our radiology department provides access to MRI scans that typically get done within < 24 h of a request, with priority for urgent admissions. Furthermore, our MRI suite has a gadolinium pump, and therefore, high-standard contrast-enhanced vascular imaging is the norm.
- More specialist personnel are available at our center compared to other cohort settings: for example, a third of the neurologists, 40% of the hematologists, and half of the neuroradiologists in the country work at our hospital.

### Study limitations

There are inherent limitations in our study:

- There is selection and sampling bias due to being a single-center and hospital-based study.
- The sample size was too small to infer any further differences between specific risk factors, for example, blood-borne virus infections.
- Our study was not designed to measure if the thrombophilia positivity in laboratory testing was further investigated and/or treated.
- We experienced attrition bias, with only 68.0% (83/122) patients coming for follow-up. This dropout rate is about average for our neurology outpatient service due to a number of factors (patients living outside the city or country, choosing a different health care provider, a broken primary health care system for monitoring, or financial constraints).

## Conclusion

Our study, the largest cohort published from the SSA, has shown similarities in demographic and clinical profiles of patients with CVST from the region. However, there were new findings such as higher-than-expected new thrombophilia diagnoses, the role of blood-borne viruses as a risk factor, and better outcomes compared to other local cohorts due to timely diagnosis and appropriate treatments. Perhaps in our setting, routinely testing for thrombophilia and blood-borne viruses to look for underlying causes of the CVST is therefore important. Finally, our study also reinforced the importance of having adequately equipped and staffed hospitals in SSA, which significantly contributed to positive outcomes.

## Data Availability

The raw data supporting the conclusions of this article will be made available by the authors, without undue reservation.

## References

[B1] Arbela P. G. D. (1964). Cavernous sinus thrombosis. East Afr Med J. 41:551–9. 14252183

[B2] Baduro Y. Ferro J. M. (2021). Cerebral venous thrombosis in Sub-Saharan Africa: a systematic review. J. Stroke Cerebrovasc. Dis. 30:105712. 10.1016/j.jstrokecerebrovasdis.2021.105712 33812172

[B3] Chepkondol G. K. Jolly P. E. Yatich N. Mbowe O. Jaoko W. G. (2020). Types and prevalence of HIV-related opportunistic infections/conditions among HIV-positive patients attending Kenyatta National Hospital in Nairobi, Kenya. Afr. Health Sci. 20, 615–624. 10.4314/ahs.v20i2.9 33163022 PMC7609085

[B4] Dadah S. M. L. Gaye N. M. Diop A. Diagne N. S. Diop M. S. Ndiaye M.. (2015). Multiple cerebral venous thrombosis revealing an HIV infection [Article in French]. Rev. Neurol. 171, 736–737. 10.1016/j.neurol.2015.05.004 26318895

[B5] Dias L. João Pinto M. Maia R. Albuquerque L. Carvalho M. (2024). Post cerebral venous thrombosis headache - prevalence, mechanisms and risk factors. J. Clin. Neurosci. 119, 205–211. 10.1016/j.jocn.2023.12.005 38141436

[B6] Duman T. Uluduz D. Midi I. Bektas H. Kablan Y. Goksel B. K.. (2017). A multicenter study of (1144). Patients with cerebral venous thrombosis: the VENOST study. J. Stroke Cerebrovasc. Dis. 26, 1848–1857. 10.1016/j.jstrokecerebrovasdis.2017.04.020 28583818

[B7] Duncan I. C. Fourie P. A. (2005). Imaging of cerebral isolated cortical vein thrombosis. AJR 184, 1317–1319. 10.2214/ajr.184.4.01841317 15788617

[B8] Ferro J. M. Bousser M. G. Canhão P. Coutinho J. M. Crassard I. Dentali F.. (2017). European Stroke Organization guideline for the diagnosis and treatment of cerebral venous thrombosis - endorsed by the European Academy of Neurology. Eur. J. Neurol. 24, 1203–1213. 10.1111/ene.13381 28833980

[B9] Ferro J. M. Canhão P. Stam J. Bousser M. G. Barinagarrementeria F. ISCVT Investigators (2004). Prognosis of cerebral vein and dural sinus thrombosis: results of the International Study on Cerebral Vein and Dural Sinus Thrombosis (ISCVT). Stroke 35, 664–670. 10.1161/01.STR.0000117571.76197.26 14976332

[B10] Ferro J. M. Coutinho J. M. Dentali F. Kobayashi A. Alasheev A. Canhão P.. (2019). Safety and Efficacy of Dabigatran Etexilate vs Dose-Adjusted Warfarin in Patients With Cerebral Venous Thrombosis: A Randomized Clinical Trial. JAMA Neurol 76, 1457–1465. 10.1001/jamaneurol.2019.2764 31479105 PMC6724157

[B11] Field T. S. Dizonno V. Almekhlafi M. A. Bala F. Alhabli I. Wong H.. (2023). Study of rivaroxaban for cerebral venous thrombosis: a randomized controlled feasibility trial comparing anticoagulation with rivaroxaban to standard-of-care in symptomatic cerebral venous thrombosis. Stroke 54, 2724–2736. 10.1161/STROKEAHA.123.044113 37675613 PMC10615774

[B12] Gaye N. M. Diagne R. Diop A. M. Ka M. Senghor A. P. Mbodji A. B.. (2021). Cerebral venous thrombosis in a sub-saharan African country: A preliminary monocentric study of a 70 case series at the neurology department of Fann teaching hospital in Dakar - Senegal. Rev. Neurol. 177, 670–675. 10.1016/j.neurol.2020.07.012 33066995

[B13] Hasford F. Mumuni A. N. Trauernicht C. Ige T. A. Inkoom S. Okeji M.. (2022). A review of MRI studies in Africa with special focus on quantitative MRI: Historical development, current status and the role of medical physicists. Phys. Med. 103, 46–58. 10.1016/j.ejmp.2022.09.016 36219962

[B14] Hunald F. A. Riel A. M. Ramorasata A. J. Tovone X. G. Andriamanarivo M. L. Rakoto-Ratsimba H. N.. (2011). Nosocomial cavernous sinus thrombophlebitis due to multidrug-resistant Staphylococcus aureus. A pediatric case report. Arch. Pediatr. 18, 529–532. 10.1016/j.arcped.2011.02.003 21420839

[B15] Idris M. N. Sokrab T. E. Ibrahim E. A. Mirgani S. M. Elzibair M. A.. (2008). Cerebral venous thrombosis. Clinical presentation and outcome in a prospective series from Sudan. Neurosciences 13, 408–411. 21063371

[B16] Kalangu K. K. (1995). Cavernous sinus thrombosis: a report of eight consecutive comatose patients. East Afr. Med. J. 722, 791–795. 8689979

[B17] Khealani B. A. Wasay M. Saadah M. Sultana E. Mustafa S. Khan F. S.. (2008). Cerebral venous thrombosis: a descriptive multicenter study of patients in Pakistan and Middle East. Stroke 39, 2707–2711. 10.1161/STROKEAHA.107.512814 18635853

[B18] Konin C. Adoh M. Adoubi A. Anzouan-Kacou J. B. Azagoh R. N'guetta R.. (2008). Unusual venous thrombosis revealing a human immunodeficiency virus infection and a protein S deficiency. Two cases and literature review. Rev. Med. Intern. 29, 508–511. 10.1016/j.revmed.2007.12.022 18304701

[B19] Lounici A. Bensefia A. Tabti E. Bestaoui M. H. (2020). A descriptive monocentric study in Algeria of adults with cerebral venous thrombosis. Rev. Neurol. 176, 614–618. 10.1016/j.neurol.2020.02.006 32334840

[B20] Makanjuola A. I. Farombi T. H. Yaria J. O. Ogunjimi L. O. Ogunniyi A. (2015). Cerebral venous thrombosis, protein S deficiency and pregnancy triad: a case report. West Afr. J. Med. 34, 201–205. 28276047

[B21] Mokgacha K. Maruza M. P. Sesay S. O. Rwegerera G. M. (2017). Cavernous sinus thrombosis in a 14-year old boy. Turk. J. Pediatr. 59, 719–723. 10.24953/turkjped.2017.06.019 30035410

[B22] Mouton J. P. Blockman M. Sekaggya-Wiltshire C. Semakula J. Waitt C. Pirmohamed M.. (2021). Improving anticoagulation in sub-Saharan Africa: what are the challenges and how can we overcome them? Br. J. Clin. Pharmacol. 87, 3056–3068. 10.1111/bcp.14768 33586223 PMC8359270

[B23] Mucyo W. Jeilan M. Varwani M. H. Ngunga M. (2022). Factors determining the use of NOACs vs. Warfarin in a Kenyan cohort of patients requiring anticoagulation. Eur. Heart J. 43:ehac544 2713. 10.1093/eurheartj/ehac544.2713

[B24] Mwita J. C. Baliki K. Tema L. (2013). Cerebral venous sinus thrombosis in HIV infected patients: report of 2 cases. Pan Afr. Med. J. 16:4. 10.11604/pamj.2013.16.4.3252 24570775 PMC3926758

[B25] Napon C. Diallo O. Kanyala E. Kabore J. (2010). Cerebral venous thrombosis in the hospital environment in Ouagadougou (Burkina Faso). Rev. Neurol. 166, 433–437. 10.1016/j.neurol.2009.09.009 19836043

[B26] Okunola P. O. Ofovwe G. E. Abiodun M. T. Azunna C. P. (2012). Superior sagittal sinus thrombosis complicating typhoid Fever in a teenager. Case Rep. Pediatr. 2012:201203. 10.1155/2012/201203 23227403 PMC3514802

[B27] Onyambu C. K. Amayo E. Kitonyi J. M. (2013). Clinical features and patterns of imaging in cerebral venous sinus thrombosis at kenyatta national hospital. East Afr. Med. J. 90, 297–304. 26862647

[B28] Onyambu C. K. Muriithi I. M. Ngare S. M. (2010). Cerebral venous sinus thrombosis: a report of two cases. East Afr. Med. J. 87, 220–224. 10.4314/eamj.v87i5.63077 23057286

[B29] Pathology and Laboratory Medicine (2015). Aga Khan University Hospital Nairobi. Available onlinew at: https://hospitals.aku.edu/nairobi/ServicesAndFacilities/Pages/PathologyandLaboratoryMedicine.aspx (Accessed 13 June, 2025).

[B30] Ruiz-Sandoval J. L. Chiquete E. Bañuelos-Becerra L. J. Torres-Anguiano C. González-Padilla C. Arauz A.. (2012). Cerebral venous thrombosis in a mexican multicenter registry of acute cerebrovascular disease: the RENAMEVASC study. J. Stroke Cerebrovas. Dis. 21, 395–400. 10.1016/j.jstrokecerebrovasdis.2011.01.001 21367622

[B31] Samuel J. Fernandes C. M. C. (1987). Lateral sinus thrombosis (A review of 45 cases). J. Laryngol. Otol. 101, 1227–1229. 10.1017/S0022215100103561 3323379

[B32] Sassi S. B. Touati N. Baccouche H. Drissi C. Romdhane N. B. Hentati F. (2017). Cerebral venous thrombosis: a tunisian monocenter study on 160 patients. Clin. Appl. Thromb Hemost. 23, 1005–1009. 10.1177/1076029616665168 27582021

[B33] Scrimgeour E. M. Neves O. Sammud M. A. (1991). Cavernous sinus thrombophlebitis in Zimbabwe. Cent. Afr. J. Med. 37, 394–397. 1806252

[B34] Seid A. B. Sellars S. L. (1973). The Management of otogenic lateral sinus disease at Groote Schuur Hospital. Laryngoscope 83, 397–403. 10.1288/00005537-197303000-00011 4690031

[B35] Sidhom Y. Mansour M. Messelmani M. Derbali H. Fekih-Mrissa N. Zaouali J.. (2014). Cerebral venous thrombosis: clinical features, risk factors, and long-term outcome in a Tunisian cohort. J. Stroke Cerebrovasc. Dis. 23, 1291–1295. 10.1016/j.jstrokecerebrovasdis.2013.10.025 24462460

[B36] Singh B. (1993). The management of lateral sinus thrombosis. J. Laryngol. Otol. 107, 803–808. 10.1017/S0022215100124478 8228594

[B37] Tadesse T. A. Tegegne G. T. Yadeta D. Chelkaba L. Fenta T. G. (2022). Anticoagulation control, outcomes, and associated factors in long-term-care patients receiving warfarin in Africa: a systematic review. Thrombosis. J. 20:58. 10.1186/s12959-022-00416-9 36192776 PMC9528137

[B38] Ulivi L. Squitieri M. Cohen H. Cowley P. Werring D. J. (2020). Cerebral venous thrombosis: a practical guide. Pract. Neurol. 20, 356–367. 10.1136/practneurol-2019-002415 32958591

[B39] Urquhart A. C. Fung G. McIntosh W. A. (1989). Isolated sphenoiditis: a diagnostic problem. J. Laryngol. Otol. 103, 526–527. 10.1017/S0022215100156786 2754325

[B40] Wasay M. Kaul S. Menon B. Dai A. I. Saadatnia M. Malik A.. (2019). Asian Study of cerebral venous thrombosis. J. Stroke Cerebrovasc. Dis. 28:104247. 10.1016/j.jstrokecerebrovasdis.2019.06.005 31350167

[B41] Zhang Y. Liu Y. Qin B. Tang S. Liang Z. (2023). Characteristics of poor prognosis in patients with cerebral venous sinus thrombosis: a multicenter retrospective study. Neuropsychiatr. Dis. Treat. 19, 1417–1426. 10.2147/NDT.S414123 37334106 PMC10276584

